# Fast Thermal Monitoring of Pulsed Laser Cleaning Processes

**DOI:** 10.3390/mi17060653

**Published:** 2026-05-25

**Authors:** Emiliia Saprykina, Jiří Martan, Denys Moskal, Milan Honner, Šimon Lintimer, Maliha Hussain, Rostislav Medlín, Petra Honnerová, Vladislav Lang

**Affiliations:** 1 Department of Machining Technology, Faculty of Mechanical Engineering, University of West Bohemia in Pilsen, Univerzitni 8, 301 00 Pilsen, Czech Republic; emiliias@fst.zcu.cz; 2 New Technologies—Research Centre, University of West Bohemia in Pilsen, Univerzitni 8, 301 00 Pilsen, Czech Republic; moskal@ntc.zcu.cz (D.M.); honner@ntc.zcu.cz (M.H.); lintis@ntc.zcu.cz (Š.L.); medlin@ntc.zcu.cz (R.M.); petrahon@ntc.zcu.cz (P.H.); vlang@ntc.zcu.cz (V.L.); 3 Department of Physics, Faculty of Applied Sciences, University of West Bohemia in Pilsen, Univerzitni 8, 301 00 Pilsen, Czech Republic; maliha@fav.zcu.cz

**Keywords:** laser cleaning, infrared thermal diagnostics, process monitoring, laser–material interaction

## Abstract

Current manual laser cleaning methods often require highly skilled operators to achieve fast and reliable cleaning. This study investigated the feasibility of measuring thermal processes during laser metal cleaning to support the development of an automated and intelligent laser cleaning system. The measurement system was based on fast infrared (IR) diagnostics and used a field programmable gate array (FPGA) for fast signal analysis. Experiments were conducted on steel substrates covered with paint, scale, or corrosion. Thermal response in real-time for every laser pulse was monitored. Paints showed no solidification plateau, while steel exhibited a clear plateau above the ablation threshold. The scaled surface showed longer time intervals and higher heat accumulation. A histogram of time intervals enabled statistical analysis of the process. Time-resolved temperature values revealed hidden processes. These findings demonstrated the potential of IR thermal diagnostics for evaluating surface conditions and providing real-time data to optimise, monitor, and control laser cleaning processes.

## 1. Introduction

Laser cleaning has established itself as an advanced, non-contact, environmentally sustainable surface treatment technology for removing oxides, corrosion products, coatings, and other contaminants from metallic and non-metallic substrates. In contrast to conventional mechanical, chemical, or abrasive techniques, laser-based cleaning enables selective material removal with minimal secondary waste and high spatial precision. The governing mechanisms are predominantly photothermal and photomechanical, and their efficiency depends critically on laser parameters—wavelength, pulse duration, fluence, repetition rate, and scanning strategy—as well as on the thermophysical properties of both the contaminant layer and the substrate. These interrelated factors define the process window within which contaminants are effectively removed while substrate modification is avoided, underscoring the importance of reliable in situ monitoring and process control [1,2,3].

The primary approaches to process monitoring of laser cleaning include laser-induced breakdown spectroscopy (LIBS) and acoustic-based methods. LIBS is the most widely studied optical technique. It works by analysing the spectral emissions generated from a laser-induced plasma to assess surface cleanliness, typically using intensity ratios of specific emission lines. This method provides non-contact measurement, high sensitivity to different cleaning states, and the potential for integration into closed-loop control systems, as demonstrated in several studies on real-time laser cleaning monitoring of metals and archaeological surfaces [4,5,6].

Acoustic monitoring methods range from simple amplitude and frequency analysis to more advanced digital signal processing techniques. These approaches detect acoustic emission signals generated during laser cleaning that correlate with changes in surface condition. Acoustic techniques also offer non-contact operation, broad applicability across different substrate–contaminant systems, and cost-effective implementation due to relatively simple instrumentation. Their effectiveness for real-time monitoring has been demonstrated through acoustic emission studies and advanced signal processing approaches such as mel-frequency cepstral analysis and spectral flux methods [7,8].

Real-time monitoring of laser-based cleaning has also been investigated using complementary diagnostic approaches: time-resolved spectroscopy [9], time-resolved imaging [10], and probe-beam reflection and laser-plume emission spectroscopy [11]. A shift from single-sensor approaches towards multi-modal and data-driven monitoring frameworks was also reported [12].

In addition to real-time in-process monitoring techniques, post-process surface characterisation methods are widely employed to evaluate the effectiveness of laser cleaning and verify substrate integrity. Surface characterisation techniques such as scanning electron microscopy (SEM), energy-dispersive X-ray spectroscopy (EDS), and 3D profilometry are commonly used to evaluate the morphological and compositional changes after laser cleaning, ensuring that the substrate remains undamaged [13,14,15,16]. However, these techniques are time consuming and do not enable process monitoring and control.

Among monitoring techniques in general, infrared (IR) thermography has emerged as a powerful non-contact method for real-time assessment of temperature fields during laser-based processes using continuous lasers. Its methodological foundation in temperature measurement and non-destructive testing provides a basis for monitoring laser-based surface treatments, particularly laser additive technologies [17,18,19,20]. However, for pulsed-laser processes, thermography is slow and not sufficiently sensitive. Measurement systems based on fast IR detectors were developed for some of these applications. Studies focusing on nanosecond-pulse laser melting and ultrashort-pulse laser micromachining revealed pronounced heat accumulation and significant temperature rises, even under processing conditions often regarded as quasi-athermal [21,22]. IR-based diagnostics enabled the identification of thermal signatures associated with the formation of laser-induced periodic surface structures (LIPSS) [23,24]. However, IR radiometry-based methods that resolve in the nanosecond time range have not yet been applied to laser cleaning.

In view of the limitations of current process-monitoring approaches and the growing demand for automation in laser cleaning, the present study addresses the need for reliable in situ diagnostics of thermal processes during pulsed-laser metal cleaning. The work focuses on real-time monitoring of IR radiation evolution during the cleaning of steel substrates and its fast real-time analysis to obtain relevant variables. By analysing the thermal response of different surface conditions, including coated, scaled, and corroded surfaces, this study evaluates the capability of fast IR diagnostics to distinguish surface states and to provide process-relevant information. The novelty of this work lies in the implementation of FPGA-based signal processing and its application in the laser cleaning process. In particular, the measurement of the time interval between the rise and fall of the signal for each laser pulse is introduced here for the first time. In addition, the analysis reveals previously unreported effects of heat accumulation during laser cleaning. The results contribute to the development of non-destructive, real-time monitoring strategies and support the advancement of automated, intelligent laser cleaning systems for industrial applications, which will likely be based on a multi-sensor approach, combining, for example, spectroscopy, acoustic, and IR radiation methods.

## 2. Experimental

The experimental setup consisted of a laser system and a measurement system. The laser system contained a pulsed fibre laser (TruPulse 3002 nano, TRUMPF, Ditzingen, Germany) with a scanning head, used as the optical excitation source. The laser operated in the nanosecond regime at a wavelength of 1064 nm. The laser beam was focused onto the sample surface using an F-theta focusing lens with a focal length of 160 mm, resulting in a spot diameter of 65 µm. The applied conditions included a hatch spacing of 0.03 mm, a scanning speed of 1500 mm/s, bidirectional scanning, and a treated area of 2 × 2 mm. Laser cleaning experiments were performed at levels below the ablation threshold (BAT) and above the ablation threshold (AAT) of the steel substrates. These regimes were chosen based on experience with the substrate materials to investigate the material response under sub-threshold conditions, where no significant substrate material removal occurs, and under super-threshold conditions, associated with significant surface ablation. This selection is consistent with reported nanosecond laser ablation thresholds for coated steels. For example, Zhu et al. [25] reported that efficient cleaning of zinc-coated HSLA steel occurs near the coating ablation threshold (~1–3 J/cm^2^), balancing material removal and substrate protection. A detailed summary of the processing parameters is provided in Table 1.

The fluence F was defined as the laser energy E per irradiated area A, expressed as $F=\frac{E}{A}$, where E is the pulse energy (J), and A is the beam spot area (cm^2^). The irradiated area A was calculated assuming a circular beam profile, where d is the spot diameter (cm): $A=\pi {\left(\frac{d}{2}\right)}^{2}$. Peak power density $P_{peak}$ was defined as the laser pulse energy E divided by the product of pulse duration τ and irradiated area A, expressed as $P_{peak}=\frac{E}{\tau \cdot A}$, where τ is the pulse duration at height 10% (s).

**Figure 1:**
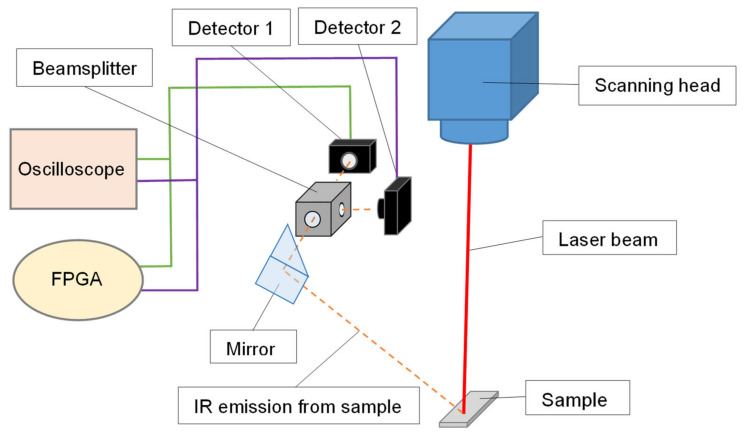
Schematic representation of the infrared measurement system based on InGaAs photodetectors with FPGA-based signal processing and oscilloscope monitoring.

The measurement system was based on fast infrared (IR) diagnostics (LabIR Ultrashort) and used a field programmable gate array (FPGA) for fast hardware signal analysis. Optical signals generated during laser irradiation were detected using two InGaAs photodetectors with a spectral range of 800–1700 nm, 5 MHz bandwidth and an active area of Ø2.0 mm (Figure 1). Long pass filters were used to cut out the laser radiation. A beam splitter divided the remaining wavelength range into two subranges: 1.25–1.5 µm for the first detector (channel C1) and 1.5–1.7 µm for the second detector (channel C2). The goal of the wavelength splitting was to evaluate temperature from the ratio of the two channels. The electrical signals from the photodetectors were recorded and analysed using a Teledyne LeCroy WavePro 404HD oscilloscope (Teledyne LeCroy, Inc., Chestnut Ridge, NY, USA), using a sampling rate of 50 MS/s. In addition, a multifunctional data acquisition instrument (FPGA) was employed for signal acquisition and fast hardware data processing. The surface condition after laser cleaning was examined using an optical microscope and scanning electron microscope (SEM) to evaluate the quality and uniformity of the cleaned area, and EDS analysis to evaluate oxide content.

All measurements were performed in three repetitions at the same place. Five places were used for statistical evaluation of results. The sample was positioned in the front of the measurement system such that the laser beam was incident from the nearly normal direction to the surface (Figure 1). The detectors observed the sample surface at a 45° angle from the normal. During laser irradiation, both detectors recorded the generated signals and simultaneously transmitted them to an oscilloscope and an FPGA-based data acquisition system connected in parallel. The acquired signals were visualised and recorded by both systems.

The signal evaluation by the FPGA-based system was done in two ways. The voltage characteristic of the detected signal, specifically the heat accumulation (HA) signal, was analysed as an average of the signal before each laser pulse. This technique was developed and implemented in-house on the FPGA. The temporal characteristic of the signal, specifically the time interval between its rising and falling edges, was analysed as the time interval duration between two defined events. The FPGA platform provided measurement results as a histogram of the number of recorded intervals. This approach enabled data collection for each repetition of the process and every laser pulse in each repetition. The configuration parameters applied to the FPGA system are summarised in Table 2. An event is defined as a single instance of signal detection by the FPGA, triggered when the input signal crosses a predefined threshold level. Each event corresponds to a time-stamped occurrence of either a rising or a falling edge, depending on the measurement channel’s configuration. The parameters were tuned to ensure a clear distinction between cleaned and uncleaned conditions.

**Figure 2:**
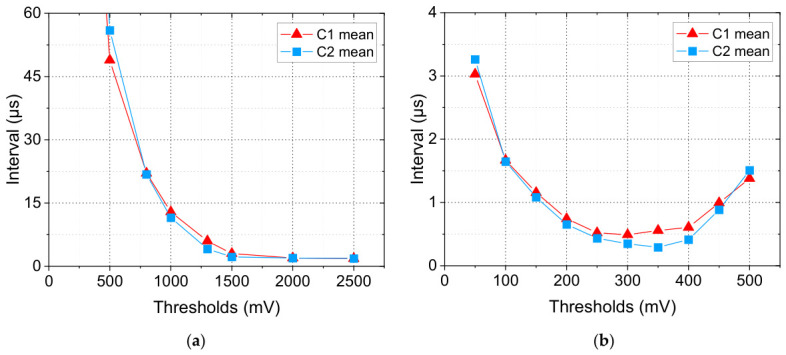
Mean time intervals for different event B (falling value) threshold settings and channels C1 and C2: (**a**) AAT parameters on S235 steel; (**b**) BAT on Max AISI 304 sample. 1x repetition only.

The rising threshold value (event A) is not critical, because the rising signal is usually very steep. The falling value (event B) setting is important because it influences the system’s sensitivity and stability. Its value was chosen at the point where the signal begins to decrease more slowly. To show the influence of the event B setting on the resulting values, a test was performed for AAT parameters on S235 steel and BAT on Max paint for the 1x repetition using different threshold settings (Figure 2). Mean time intervals are shown. The mean time interval is the weighted average of the time intervals between consecutive events, with each interval weighted by its number. It is obtained by dividing the sum of all measured intervals by their total number and is used as a characteristic value describing the typical temporal characteristics of the laser process.

The setting of the falling threshold value (event B) significantly influences the resulting mean time interval (Figure 2). For AAT parameters, the function decreases with increasing threshold. For BAT, the function has a minimum. For AAT, when the threshold is too high (>2 V), the mean interval is not sensitive to the process and is close to the detector fast decreasing curve from saturation. On the other hand, when the threshold value is set too low (<0.7 V), a certain number of intervals starts to be longer than one period between laser pulses (50 µs for AAT) due to significant HA and its variations; therefore, the best window is between 0.8 and 1.5 V. For BAT, the phenomenon of longer intervals than period (3.5 µs for BAT) happens for both small and high thresholds, because the signal peaks are not so high (500–1000 mV) and HA is relatively high (50–200 mV); therefore, the middle values between 200 and 400 mV are recommended.

**Figure 3:**
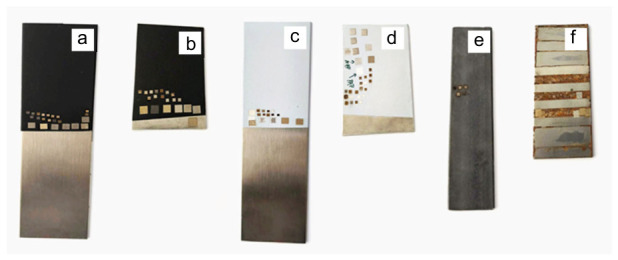
Samples used for the experimental measurements: (**a**) Max AISI 304; (**b**) Max S235; (**c**) Body AISI 304; (**d**) Body S235; (**e**) Scale S235; (**f**) Corrosion S235. “Max” corresponds to black paint, while “Body” corresponds to grey paint.

Six representative samples (Figure 3) were selected in order to investigate the thermal response and cleaning behaviour of different steel substrates and surface conditions during laser cleaning. Samples 1 and 3 were based on AISI 304 stainless steel, while samples 2, 4, 5, and 6 were prepared from S235 structural steel. The sample dimensions varied: length ranged from 7.5 to 20 cm, width from 3 to 5.2 cm, and thickness from 1.3 to 3 mm.

Painted samples consisted of steel substrates covered with an industrial paint layer, representative of typical protective coatings used in industrial environments: BODY P360 2K HS PRIMER GREY (Industrial Area Sindos, P.C., Thessaloniki, Greece) and SprayMax 1K UNIFILL S7 (Peter Kwasny GmbH, Gundelsheim, Germany). In contrast, the Scale and Corrosion samples were unpainted and characterised by naturally or artificially formed oxide and corrosion layers. The average thickness of the Max paint coating was approximately 38 µm on AISI 304 stainless steel and 33 µm on S235 structural steel. In contrast, the Body paint coating exhibited an average thickness of about 64 µm on AISI 304 and 65 µm on S235 steel. Additionally, the corrosion layer formed on S235 steel had an average thickness of approximately 21 µm.

The chemical composition (wt.%) of AISI 304 stainless steel and S235 structural steel is presented in Table 3. AISI 304 is an austenitic stainless steel characterised by high corrosion resistance, whereas S235 is a non-alloy structural steel known for its good weldability and mechanical performance. The compositional data are provided in accordance with ASTM A240, EN 10088-2, and EN 10025-2 [26,27,28].

## 3. Results

### 3.1. Oscilloscope Results

**Figure 4:**
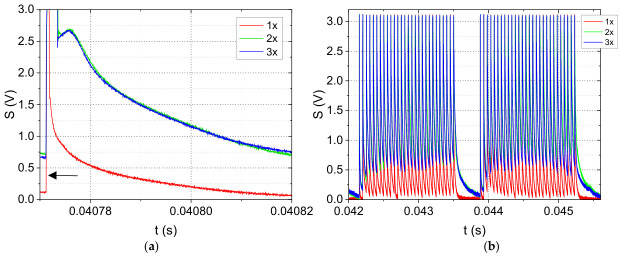
Time-domain response of the Max AISI 304 sample under AAT conditions: (**a**) zoomed-in view of the C1 channel response showing 1x, 2x, and 3x responses to a single laser pulse, the arrow indicating the beginning of the laser pulse; (**b**) full temporal response over two consecutive laser pulse sequences, demonstrating the signal shape in two scanned laser lines.

Figure 4 presents the time-domain response of the Max AISI 304 sample processed above the ablation threshold (AAT). Here, S denotes the signal amplitude, and t represents time. The experiment was performed in three consecutive repetitions (1x, 2x, and 3x), and the data were recorded from the first measurement channel (C1). C1 and C2 denote the two input channels of the oscilloscope, corresponding to detector 1 and detector 2, respectively. Each channel displays the signal from its detector, and the difference between them comes from the wavelength range that each detector is sensitive to.

In the zoomed-in view (Figure 4a), a pronounced difference is observed between the first and subsequent repetitions. During the first pass, the signal reaches 3.1 V (the detector’s saturation limit) and rapidly decays to below 0.1 V before the next pulse arrives. This corresponds to laser interaction with and removal of the paint layer. In contrast, during the second and third passes, the post-peak signal stabilises at a significantly higher level (2.7 V) and gradually decreases to 0.7 V. This stabilised value is called a plateau and is caused by the production of latent heat during the solidification phase change in the material. The similarity of the 2x and 3x responses confirms effective coating removal during the 1x pass under AAT conditions.

The full temporal response shown in Figure 4b presents two consecutive laser pulse sequences recorded in the time interval of approximately 0.042–0.045 s. The two sequences result from two lines of laser pulses incident on the material surface. The signal consists of a series of sharp peaks corresponding to individual laser pulses. The heat accumulation signal is low for the 1x repetition (0.1 V), but significantly increases for 2x and 3x repetitions (0.7 V). Between the pulse sequences, the signal decreases to near the baseline level of 0–0.1 V. The higher heat accumulation is also caused by the solidification phase change, which prolongs the time of resting at high temperature.

**Figure 5:**
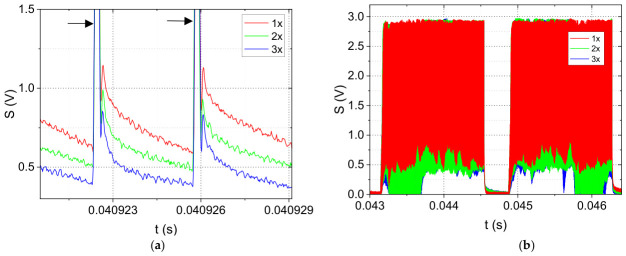
Time-domain response of the Scale S235 sample under BAT conditions: (**a**) zoomed-in view of the C1 channel response showing 1x, 2x, and 3x events for two consecutive laser pulses, the arrow indicating the beginning of the laser pulse; (**b**) full temporal response illustrating two consecutive laser pulse sequences, demonstrating the repetitive back-and-forth scanning behaviour and removal of scale in the areas of low heat accumulation.

Figure 5 presents the time-domain response of the Scale S235 sample processed below the ablation threshold (BAT). The measurements were performed in three consecutive repetitions (1x, 2x, 3x). In the zoomed-in view (Figure 5a), the responses for all three repetitions exhibit similar peak amplitudes and decay behaviour. The signal reaches its saturated maximum, then decays gradually. After the peak, the amplitude decreases to about 0.9–1.1 V for 1x, 0.6–0.7 V for 2x, and 0.4–0.5 V for 3x repetition, and continues to decline smoothly without abrupt changes. No significant increase in post-peak signal level is observed for subsequent passes. The short oscillation after the peak is caused by the detector characteristics.

The full temporal response shown in Figure 5b illustrates two consecutive laser pulse sequences (lines on the sample). The laser can be observed scanning back and forth during the filling process. In the 1x repetition, the heat accumulation (HA) signal (lower envelope of the signal) is oscillating at high values between 0.4 and 0.8 V. For the 2x and 3x repetitions, the HA signal had two distinct parts. The first part was stable at almost 0.0 V value, and the second was slightly changing around 0.4 V. The first part is a signal of already cleaned scale from the surface, so of the steel substrate. The second part is a signal of the remaining scale on the surface. The microscope image (see below) confirms that the scale is only partially removed by the BAT process, even after 3x repetition. The increased HA can thus clearly show the location of the not-yet-removed scale.

**Figure 6:**
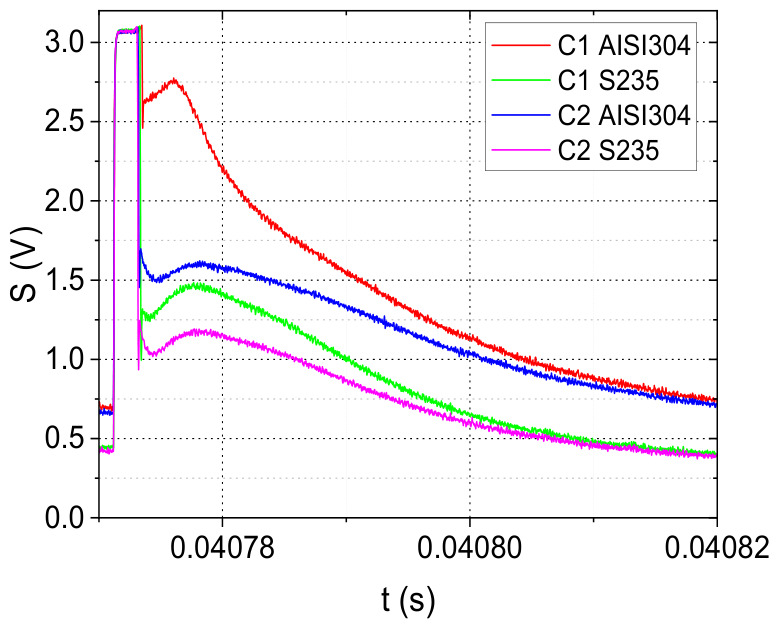
Comparison of time-domain signal responses for two tested materials, AISI 304 stainless steel and S235 structural steel, obtained during the third repetition of the AAT process. The signals were recorded using two detectors corresponding to channels C1 and C2. The presented data illustrate the temporal behaviour of the signals and highlight differences in response characteristics between the two material samples under identical testing conditions.

Figure 6 presents the time-domain response recorded during 3x repetitions of AAT laser processing for Body paint applied to AISI 304 and S235 steels. The signals from both measurement channels (C1 and C2) are shown for comparison.

For both steels, a solidification plateau (a temperature range during which the material remains nearly constant) is observed after the saturated peak signal. For channel C1, the AISI 304 sample signal reaches the plateau level of approximately 2.75 V, whereas S235 exhibits a lower plateau level of about 1.45 V. In channel C2, the corresponding levels are reduced to approximately 1.6 V for AISI 304 and 1.2 V for S235. The length of the plateau is very similar for the two materials, but the shape of the curves is very different, mainly for C1. From the ratio of the two channels, temperature can be calculated [29].

Temperature is estimated using the two-colour pyrometry method based on Planck’s law of thermal radiation. By measuring the thermal radiation of an object within two spectral bands ($\lambda_{1,min}-\lambda_{1,max},\;\lambda_{2,min}-\lambda_{2,max}$), two signal intensities, I1 and I2, are determined, and the following intensity ratio r can be determined: $r=I_{1}/I_{2}=\frac{\int_{\lambda_{1,max}}^{\lambda_{1,max}}\eta_{1}\cdot M_{\lambda ,1}(\lambda_{1,}T)\cdot d\lambda }{\int_{\lambda_{2,max}}^{\lambda_{2,max}}\eta_{2}\cdot M_{\lambda ,2}(\lambda_{2,}T)\cdot d\lambda }$, where η is the combined efficiency of the optical path and the quantum efficiency of the detector, and M is spectral radiance. The temperature T, expressed independently of emissivity, is given by: $T=\frac{k_{B}\cdot \left(\lambda_{1}-\lambda_{2}\right)}{\lambda_{1}\cdot \lambda_{2}\cdot ln{\left(r\cdot \frac{\lambda_{1}}{\lambda_{2}}\right)}^{5}}$.

An experimental calibration of the two-colour pyrometry system was performed using a reference source with known temperatures. The ratio of the detected signals was measured over a range of temperatures, and a calibration curve relating the intensity ratio to temperature was obtained. This calibration accounts for the spectral response of the optical system and detector sensitivities and was used for subsequent temperature determination.

**Figure 7:**
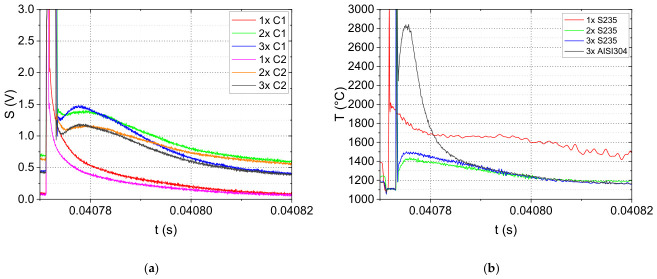
(**a**) Comparison of time-domain responses of sample Body S235 for AAT process in channels C1 and C2 for different repetitions; (**b**) temperature evolution during three repetitions of the AAT process for the Body S235 steel sample. The AISI 304 substrate is included for comparison purposes. The presented results show the time evolution of temperature throughout the process and enable comparison of the thermal response between the S235 sample and the AISI 304 substrate under identical experimental conditions.

A representative result is shown in Figure 7b. The unexpected result is that the ratios are very different for the two materials: for one steel, the solidification temperature is around 1450 °C (S235); for the second, it is 2800 °C (AISI 304), which does not seem correct. This temperature is close to the boiling point of Fe (2861 °C), Cr (2671 °C) and Ni (2913 °C) [30].

The difference between the two steels could also be caused by spectral emissivity differences affecting the ratio or by plasma emissions. The emissivity was measured in the spectral range from 1.5 to 1.7 µm (Figure S11), and the resulting mean values in this range are 0.35 for AISI 304 and 0.34 for S235—very similar results. Further, it is not expected to change significantly in the second wavelength range. On the other hand, plasma emission can persist for several microseconds with a nanosecond laser [31]. The emissivity of Body and Max paints and Scale was found to be high (0.85–0.97).

Following the peak, all signals exhibit a gradual decay. After 50 µs of cooling, the signals decrease to approximately 0.7 V for AISI 304 and 0.4 V for S235 steels. At this time, both channels are equal for each material. It means the same temperature for both materials.

Despite the differences in peak intensity, the temporal profiles show similar decay behaviour, indicating comparable post-pulse cooling dynamics. The similarity in signal shape confirms that during the third repetition, the coating had already been removed and the laser was interacting directly with the metallic substrate.

Figure 7b shows the temporal evolution of the surface temperature of the Body S235 sample during three repetitions under AAT conditions. In the 1x repetition, the temperature reaches 2000 °C during the rapid heating phase, and then gradually decreases to around 1660 °C, where a long plateau appears. This temperature is very close to the melting point of titanium (1668 °C). According to the producer, the Body spray contains a low amount (1 to 10%) of TiO2 particles. EDS analysis of the paint revealed 5.8% (wt.%) of Ti before laser irradiation (Figure S12) and 0.5% after 1x repetition at AAT conditions (Figure S13). After 3x repetition, there is only 0.1% of Ti remaining at the surface. Therefore, it seems that the plateau at the 1x repetition is caused by solidification of metallic Ti dissociated from TiO2 by the laser beam. It is interesting that this plateau appears only in the temperature evolution and not in the signal evolution. (Figure 7a). This means that only a small amount of material solidifies. The 2x and 3x repetitions show a lower maximum temperature, peaking between 1400 and 1500 °C, before steadily cooling down to approximately 1180 °C. The peak temperature is observed 4 µs after the laser pulse and about 2 µs before the peak of the C2 channel plateau signal in the 2x and 3x repetitions (Figure 7a).

**Figure 8:**
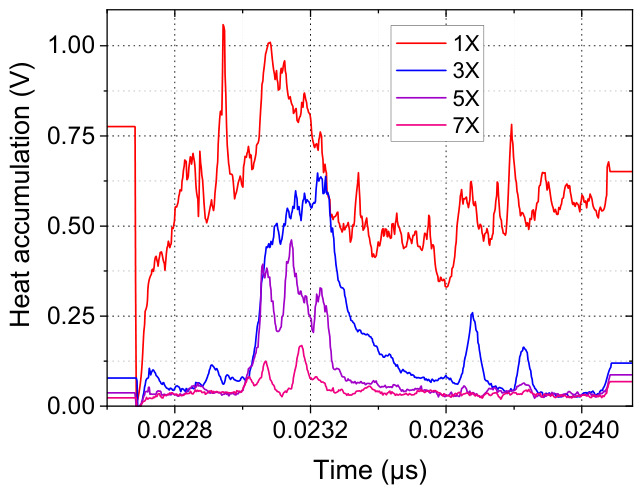
Comparison of heat accumulation signal responses obtained by FPGA fast analysis for Scale S235 sample during the 1x–7x repetitions of the BAT process on the selected line of scanning in the middle of the sample. The data demonstrate a gradual process of scale surface cleaning. A decrease in heat accumulation reflects the removal of scale. In certain places, parts of the scale remain even after 7x repetitions.

To evaluate the efficiency of scale removal, the evolution of heat accumulation (HA) was analysed as a function of processing time for different numbers of passes (Figure 8). A high HA signal indicates laser interaction with scale, while a low signal indicates interaction with the steel substrate. A stable, low HA signal indicates complete scale removal.

At a single pass (1x), the signal remains high and fluctuating with significant amplitude variations, indicating incomplete and non-uniform scale removal. With an increasing number of passes (3x and 5x), the signal shows a gradual reduction in peak intensity and variability. This suggests progressive removal of the scale layer, although some irregularities persist. After seven passes (7x), the curve becomes nearly flat with minimal fluctuations, demonstrating a stable thermal response. This behaviour corresponds to an almost complete removal of the scale. The results clearly indicate that increasing the number of passes significantly improves the efficiency of scale removal.

### 3.2. FPGA Results

**Figure 9:**
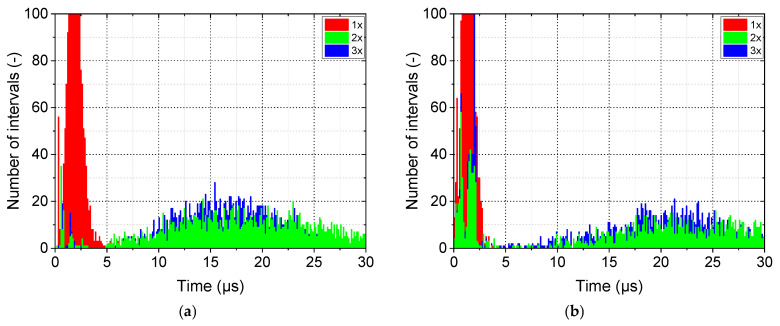
Histograms of infrared (IR) radiation time intervals recorded during the AAT process for the Body AISI 304 sample, channel C1 (**a**) and channel C2 (**b**). The distribution of time intervals reflects the temporal characteristics of thermal processes after laser pulses, providing insight into their frequency and variability.

The IR radiation time interval results for the Body AISI 304 sample, obtained from the FPGA, are shown in Figure 9. This figure shows the distribution of time intervals between rising and falling signal events. Channel 1 (a) indicates that, for the 1x repetition, the signal is mainly characterised by short intervals in the range of 0–5 µs, reaching counts of up to 180, which are related to the paint-removal phase. For 2x and 3x repetitions, a broader spread of longer intervals between 6 and 25 µs emerges, although with lower counts of up to 28. These longer intervals correspond to the metal solidification plateau and suggest a shift from coating ablation to direct interaction with the AISI 304 substrate. Channel 2 (b) shows that, for the 1x repetition, short intervals of 0–3 µs dominate, with counts up to 200 corresponding to the paint-removal stage. For 2x and 3x repetitions, additionally, a wide distribution of intervals appears at longer values of 10–30 µs, with a lower count up to 20.

**Figure 10:**
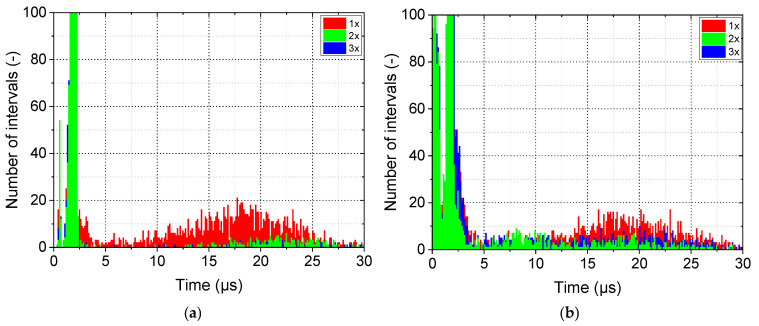
Histograms of infrared (IR) radiation time intervals recorded during the AAT process for the Corrosion S235 sample, channel C1 (**a**) and channel C2 (**b**). The distribution of time intervals captures the temporal behaviour of the thermal processes after laser pulses, providing insight into their frequency and variability.

The results for the Corrosion S235 sample are shown in Figure 10. Channel C1 (a) reveals that, during the 1x repetition, short intervals within the 0–3 µs range occur less frequently than in the 2x and 3x repetitions. In contrast, a broader distribution of longer intervals between 10 and 25 µs is observed, with counts reaching up to 21. These results imply that part of the corrosion layer had already been removed after the first pass. Channel C2 (b) similarly demonstrates that for the 1x repetition, the number of short intervals in the 0–4 µs range is lower compared to the 2x and 3x repetitions. However, a broader distribution of intervals appears at longer values (14–24 µs), with a count up to 18 µs.

**Figure 11:**
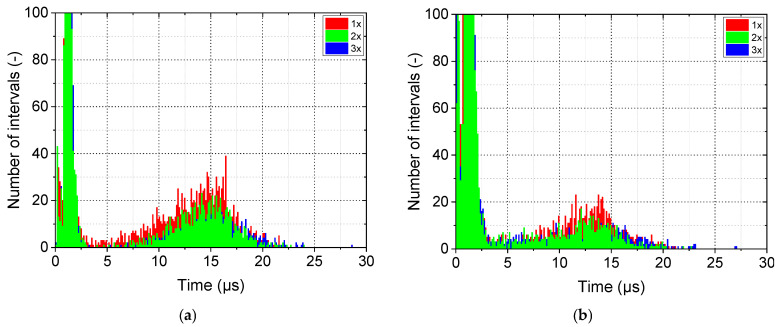
Histogram of infrared (IR) radiation time intervals recorded during the AAT process for the Scale S235 sample, channel C1 (**a**) and channel C2 (**b**). The distribution of time intervals characterises the temporal behaviour of thermal processes after laser pulses, providing insight into their frequency and variability.

The results for the Scale S235 sample are shown in Figure 11. Channel C1 (a) indicates that, across all three repetitions, the number of short intervals within the 0–3 µs range remains nearly constant. In addition, a broad distribution of longer intervals between 7 and 18 µs is observed, with counts reaching up to 39. Channel C2 (b) reveals that, for all three repetitions, the number of short intervals in the 0–3 µs range is almost the same. A wide distribution of intervals is observed at longer values, 8–15 µs, with counts up to 23 µs. Over time, the number of intervals declines, suggesting a decrease in the frequency of detected events with longer intervals as the process continues.

To improve the reliability of the results, the experiment was repeated at five locations, with three laser cleaning repetitions performed at each location. The obtained data showed variations between measurements due to external factors and measurement errors. To quantitatively assess the spread of the results, the standard deviation was used. This statistical measure characterises how much individual measurements deviate from the mean and allows evaluation of the stability and repeatability of the experiment.

**Figure 12:**
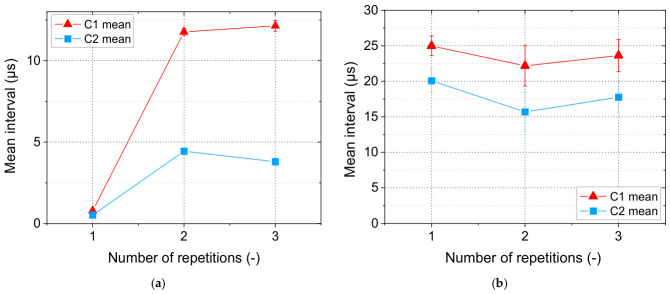
Variation of IR radiation interval mean values and standard deviation during the AAT process repetitions for the (**a**) Max AISI304 sample and (**b**) Corrosion S235 sample. Error bars represent standard deviation.

Under the AAT conditions (Figure 12), for the Max AISI 304 sample (a), the interval values increased sharply after the 1x repetition. The mean interval of channel C1 rose from 1 μs to 13.5 μs in the 2x repetition and slightly increased to 14.5 μs in the 3x. Channel C2 increased from 0.75 to 4 μs in the 2x repetition and decreased to 3.8 μs in the 3x. The standard deviation remained small, implying that the process was stable.

In the case of the Corrosion S235 sample (Figure 12b), relatively high intervals were already observed in the 1x repetition (25 μs for C1 and 20 μs for C2). With increasing repetitions, the mean intervals gradually decreased to 22 μs (C1) and 16 μs (C2) in the 2x repetition and increased to 23.75 μs (C1) and 17.5 μs (C2) in the 3x repetition. The Corrosion sample showed greater fluctuations than the Max AISI 304 sample.

**Figure 13:**
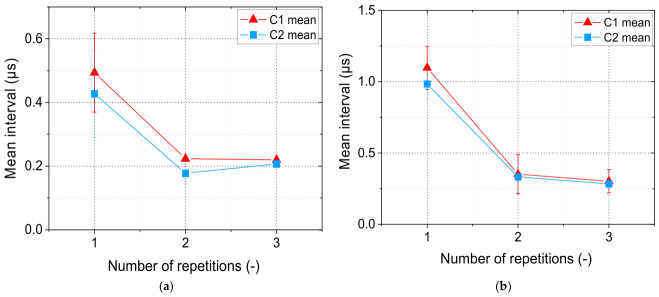
Variation of IR radiation interval mean values and standard deviation during the BAT process repetitions for the (**a**) Max AISI304 sample and (**b**) Corrosion S235 sample. Error bars represent standard deviation.

Under the BAT conditions (Figure 13), regarding the Max AISI 304 sample (a), the mean interval of channel C1 decreased sharply from 0.5 μs in the 1x repetition to 0.22 μs in the 2x and remained nearly constant (0.21 μs) in the 3x repetition. The mean interval C2 decreased from 0.42 μs to 0.17 μs and increased to 0.20 μs. The BAT process for the AISI304 sample demonstrated a clear reduction in variability with increasing repetitions, indicating progressive process stabilisation.

For the Corrosion S235 sample (Figure 13b), the mean interval of channel C1 decreased from 1.12 μs to 0.37 μs, while channel C2 decreased from 1.0 μs to 0.35 μs in the 2x repetition and continues to decrease to 0.37μs (C1) and 0.35 μs (C2) in the 3x repetition. The Corrosion S235 sample also showed a decreasing standard deviation across repetitions, but maintained higher variability than the Max AISI 304 sample.

### 3.3. Surface Characterisation

**Figure 14:**
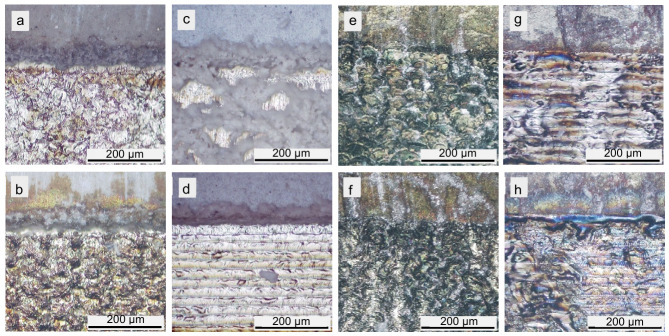
Comparison of surface morphology of Body AISI 304 and Scale S235 samples after AAT and BAT processing with 1x and 3x repetitions. Panels (**a**–**d**) show the AISI 304 sample, while (**e**–**h**) show the Scale S235 sample. Specifically, (**a**,**e**) AAT—1 repetition, (**b**,**f**) AAT—3 repetitions, (**c**,**g**) BAT—1 repetition, and (**d**,**h**) BAT—3 repetitions. In each image, the upper region represents the initial surface of the sample before treatment, while the lower region shows the laser-processed area. Scale bar: 200 µm.

To further validate the signal-based analysis, the processed surfaces were examined using optical microscopy. The resulting microstructural observations (Figure 14) reveal clear differences in surface morphology, depending on the processing regime and the number of repetitions.

In the case of the Body AISI 304 sample (Figure 14a–d), the AAT treatment leads to visible surface modification already after the first repetition (Figure 14a), where partial melting and irregular microstructures appear near the treated region. After three repetitions (Figure 14b), the modified zone becomes more pronounced, showing a thicker transformed layer and a more heterogeneous microstructure, indicating increased thermal interaction and material restructuring and remelting. Regarding the BAT conditions (Figure 14c–d), the surface modification is negligible after a single repetition (Figure 14c), but residuals of the paint are still present on the steel surface. After three repetitions (Figure 14d), the paint is almost completely removed and parallel slightly remelted features appear, reflecting cumulative thermal effects during repeated irradiation.

For the Scale S235 sample (Figure 14e–h), the AAT process produces noticeable surface modification already after the first repetition (Figure 14e), characterised by a rough and irregular morphology associated with the disruption of the scale layer. After three repetitions (Figure 14f), the modified region becomes more uniform, indicating progressive removal and restructuring of the oxide scale. Under BAT conditions (Figure 14g–h), the surface retains more pronounced layered features related to the original scale structure. After one repetition (Figure 14g), parallel patterns corresponding to the laser scanning direction are visible. After three repetitions (Figure 14h), the treated region becomes more heterogeneous, showing partial removal of the oxide scale on isolated areas.

**Figure 15:**
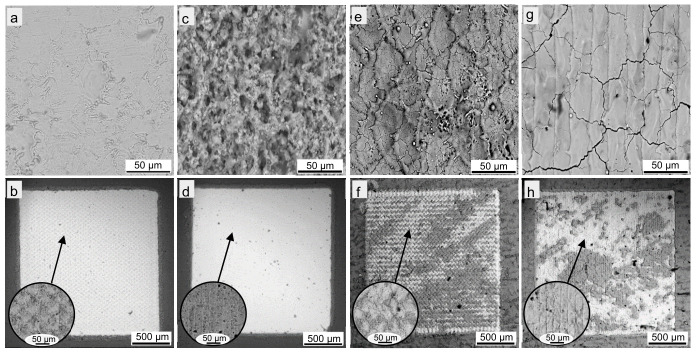
SEM images of Body S235 and Scale S235 samples after AAT and BAT processing with one and three repetitions. Panels (**a**–**d**) show the Body S235 sample, while (**e**–**h**) show the Scale S235 sample. Specifically, (**a**,**e**) AAT—1 repetition, (**b**,**f**) AAT—3 repetitions, (**c**,**g**) BAT—1 repetition, and (**d**,**h**) BAT—3 repetitions. The circled region indicates an area of interest, shown in higher magnification in the inset (50 µm scale bar). Main images scale bar: (**a**–**g**) 50 µm, (**b**–**h**) 500 µm.

Figure 15 shows SEM BE (Backscattered Electron) images of S235 steel samples after laser cleaning, comparing two different surface conditions: the base material paint and the oxide layer. For each material, two laser cleaning regimes were applied—AAT and BAT—with either one repetition or three repetitions. The top-row images are shown at higher magnification (50 µm scale bar) to highlight fine surface features, whereas the bottom-row images are presented at lower magnification (500 µm), with inset images providing detailed views of selected regions.

The Body S235 sample (Figure 15a–d) exhibits significant coating removal after a single pass under AAT conditions (Figure 15a), with the paint layer largely eliminated (95%). At the same time, localised melting of the metallic substrate is observed, but the substrate is only slightly damaged (resolidified droplets, material reflow). After three passes (Figure 15b), the coating is almost completely removed (98%), with only minor residual traces remaining at isolated locations. The treated surface exhibits significantly pronounced remelting and morphological modification by ablation. Under BAT conditions (Figure 15c–d), the cleaning efficiency is significantly reduced. After a single pass (Figure 15c), the paint layer is ablated from surface and stays open. After three passes (Figure 15d), most of the coating is eliminated (96%). Localised paint residuals persist.

As shown for the Scale S235 sample (Figure 15e–h), AAT processing leads to noticeable surface modification after the first pass (Figure 15e), characterised by surface melting and crack formation associated with thermally induced stresses; however, the scale is not removed (1% removal). After three passes (Figure 15f), the treated region remains only partially cleaned (27%), while more pronounced melting of the substrate is evident. Under BAT conditions (Figure 15g–h), the surface shows minimal modification. After a single pass (Figure 15g), the oxide scale remains largely intact, with slight melting and the formation of relatively large cracks. After three passes (Figure 15h), half of the scale is already removed (better than AAT) and the surface retains a heterogeneous morphology with persistent oxide residues.

To quantify the degree of surface cleaning after laser treatment, a grid-based image analysis method was employed. A 10 × 10 square grid was overlaid on optical micrographs of the treated area (2 × 2 mm). Each grid cell was evaluated individually and classified according to the extent of surface cleaning. If the entire cell area appeared cleaned by the laser, it was marked as “clean.” If the surface within a cell remained insufficiently cleaned, it was marked as “unclean.” In cases of partial cleaning, the cell was considered “clean” when at least 50% of its area showed effective cleaning. Using this approach, the overall percentage of cleaned surface area was determined from the micrographs (Table 4).

The results summarised in Table 4 demonstrate that processing at fluences above the ablation threshold provides significantly higher cleaning efficiency compared to below-threshold conditions for most of the investigated materials. In most cases, near-complete or complete coating removal (95–100%) is achieved already after a single pass, with full cleaning typically reached after three passes. In contrast, processing below the ablation threshold results in substantially lower cleaning efficiency, particularly after a single pass, where only limited coating removal is observed (as low as 1–8% for Body S235 and Body AISI 304). Although increasing the number of passes significantly improves the cleaning performance under these conditions, some small residual contamination often remains. The Scale S235 sample exhibits the lowest cleaning efficiency under both processing regimes, indicating the higher resistance of oxide scale to laser removal. Even after three passes, the cleaning degree remains relatively low (27% and 53% for AAT and BAT conditions, respectively), highlighting the difficulty of removing this type of surface layer.

In addition to evaluating the cleaning efficiency, the effect of laser treatment on surface topography was assessed through roughness measurements. Surface roughness was measured using an Olympus laser confocal profilometer in directions parallel and perpendicular to the laser scanning direction. The results are summarised in Table 5.

The surface roughness measurements presented in Table 5 demonstrate a pronounced influence of both laser processing parameters and coating type on the resulting topography of AISI 304 and S235 steels. In addition, scale and corrosion are associated with relatively high roughness values, reflecting their inherently heterogeneous and irregular structure. Laser cleaning at 1x repetition under the AAT process did not significantly change the surface roughness in most cases. On the other hand, 3x repetition of the AAT process resulted in a significant increase in surface roughness, where Ra values of 3–4 μm were frequently attained. This trend suggests that the AAT induces not only coating removal but also noticeable modification of the substrate. The BAT processing produced lower roughness values in most cases, indicating soft substrate material remelting or polishing, when coating or corrosion was removed. The type of coating played a critical role in the cleaning outcome. The Max coating was removed relatively uniformly, whereas the Body coating showed less consistent behaviour. Notably, for Body coating treated under BAT conditions at 1x repetition, an anomalous increase in roughness was observed, because of incomplete coating removal. Additionally, the comparison between measurements parallel and perpendicular to the laser scanning direction (${Ra}_{∥}$ and ${Ra}_{⊥}$) indicates the development of surface anisotropy. This effect becomes more pronounced with increasing processing intensity, suggesting the formation of directional surface features aligned with the laser path.

## 4. Discussion

The results demonstrate that the AAT laser cleaning regime produces significantly longer IR radiation intervals due to a significant amount of melt, whereas the BAT regime is characterised by short intervals (Figures S1–S6). For both regimes, however, the newly developed IR radiation measurement and fast analysis give a clear distinction between a not-cleaned and a cleaned surface. For the AAT regime, the dominant factor influencing the IR observations is the amount of melt, although the substrate material is also ablated, not only melted. The effect of the cleaning process becomes apparent in subsequent repetitions. The key observation is whether the removed material corresponds to the coating or the substrate, rather than the absolute removal depth. The main difference lies in longer time intervals and greater heat accumulation in the steel substrate, due to significant melting.

In the BAT regime, the dominant factor is the surface material’s thermal conductivity, although the substrate material is also melted, not only heated. Paint, scale, and corrosion layers exhibit lower thermal conductivity than the substrate; therefore, the time intervals and heat accumulation decrease once the substrate is reached. This transition may occur gradually across successive repetitions (e.g., in the case of a scale), leading to a corresponding gradual decrease in the mean interval. Such behaviour could potentially be calibrated for specific material combinations.

The AAT parameters effectively remove the paints already at 1x repetition, but significantly damage the substrate surface at 3x repetition. On the other hand, BAT parameters remove the paint more slowly, but do not significantly damage the material. Moreover, the steel surface is polished to lower roughness by soft remelting after 3x repetition.

The emissivity of most samples was measured (Figure S11). The emissivity of Body and Max paints and Scale was found to be high (0.85–0.97). The emissivity of steel substrates was found to be in the middle range (around 0.35). This means a value that is more than 2 times lower. Despite this fact, the cleaning was detected from the measured values. For paints and AAT parameters, there was a significant increase (10 times) in the mean time interval, and for the Scale at BAT, there was a significant decrease (10 times) in heat accumulation.

One calibration setting for temperature determination (emissivity ratio) was used for all samples. Despite this, the solidification plateau for Ti was found to be in perfect agreement with its melting point (1668 °C), and the plateau value for steel S235 was very close to its solidus temperature (1430 °C). Moreover, the temperatures for both steel substrates at 3x repetition are almost identical and follow the same time evolution, starting at 15 µs after the laser pulse, despite the different signal values.

## 5. Conclusions

The first experimental investigation in the literature of laser cleaning of steel surfaces using fast IR detectors and an FPGA-based real-time analysis was conducted. It showed that distinct differences can be observed between the first laser pass and subsequent passes. This is particularly pronounced on the painted surfaces, indicating that the paint has been removed. From the raw signals, the time intervals between the signal rise and fall and the heat accumulation signal were analysed in real time for each laser pulse. From the ratio of two detector signals, the temperature was calculated.

For painted surfaces and the laser process above the ablation threshold (AAT), a second broad maximum in the time intervals during the second pass appeared and so the mean time interval increased. The second maximum corresponded to a solidification plateau, reflecting the exposure of the steel substrate. Further, heat accumulation increased significantly after the paint was removed. For scale and corrosion layers, variations between passes were less pronounced. At lower pulse energies (BAT), the plateau was absent in the signal, suggesting minimal substrate melting. The removal of coatings, scale or corrosion was detected by a decrease in mean time interval. The partial removal of scale was also detected by the change in heat accumulation during a single line of pulses. Overall, the mean time interval and heat accumulation signal showed to be the most significant characteristics of laser cleaning performance, although other parameters can be also determined (e.g., temperature or histogram of time intervals) and used in future evaluation by AI/ML approaches.

The FPGA enabled real-time statistical evaluation of signal shape after every laser pulse. With proper threshold settings, there is no need for manual evaluation of the time-resolved data. The threshold setting was also discussed. The results demonstrate that fast IR monitoring is sensitive to surface changes and suitable for diagnostic applications, automated FPGA data evaluation, and future implementation in industrial laser cleaning systems for automated laser processing, potentially using AI/ML-based analysis and multi-sensor fusion.

## Figures and Tables

**Table 1:** Laser processing parameters for nanosecond pulses.

Process	Frequency of Pulses [kHz]	Waveform [-]	Pulse Energy [mJ]	Pulse Width [ns]	Fluence [J/${cm}^{2}$]	Power Density [W/${cm}^{2}$]
AAT	20	32	1.00	220	30.1	1.37·$10^{8}$
BAT	290	28	0.07	10	2.11	2.11·$10^{8}$

**Table 2:** Parameters set for the time intervals evaluation by the FPGA system.

	AAT		BAT	
Event	A	B	A	B
Edge	Rising	Falling	Rising	Falling
Threshold	2.000 V	1.300 V	300 mV	250 mV

**Table 3:** Chemical Composition of Materials (wt.%).

AISI 304 Stainless Steel Typical Composition According to ASTM A240/EN 10088 [26,27]		S235 Structural Steel Typical Composition According to EN 10025-2 [28]	
Element	Content (wt.%)	Element	Content (wt.%)
Iron (Fe)	Balance	Iron (Fe)	Balance
Chromium (Cr)	18.0–20.0	Carbon (C)	≤0.17
Nickel (Ni)	8.0–10.5	Manganese (Mn)	≤1.40
Carbon (C)	≤0.08	Silicon (Si)	≤0.05
Manganese (Mn)	≤2.0	Phosphorus (P)	≤0.025
Silicon (Si)	≤1.0	Sulfur (S)	≤0.025
Phosphorus (P)	≤0.045	Nitrogen (N)	≤0.012
Sulfur (S)	≤0.030		

**Table 4:** Material cleaning summary table (%).

Cleaned (%)				
Sample	AAT		BAT	
	1x	3x	1x	3x
Max AISI 304	100	100	70	98
Max S235	99	100	66	94
Body AISI 304	99	100	8	96
Body S235	95	98	1	96
Scale S235	1	27	7	53
Corrosion S235	98	100	80	96

**Table 5:** Surface roughness of materials before and after laser process.

Surface Roughness Ra [μm]							
Sample	Direction	Steel	Sample Before Processing	AAT		BAT	
				1x	3x	1x	3x
Max AISI 304	‖	0.273	1.246	1.127	4.013	0.726	0.737
	⊥	0.215	1.022	1.079	4.029	1.049	0.741
Max S235	‖	0.264	1.689	1.131	3.103	0.698	0.381
	⊥	0.366	0.985	0.609	2.208	0.925	0.993
Body AISI 304	‖	0.252	0.545	0.948	3.043	8.259	0.446
	⊥	0.259	0.607	1.064	3.197	10.756	0.75
Body S235	‖	0.396	0.594	0.795	1.931	5.340	0.596
	⊥	0.294	0.792	0.910	2.207	6.895	0.761
Scale S235	‖	-	1.133	1.601	2.038	1.370	2.159
	⊥	-	0.819	1.209	1.505	1.474	2.396
Corrosion S235	‖	-	1.245	1.473	2.960	0.632	0.448
	⊥	-	1.452	1.820	2.576	0.719	0.67

Symbols ‖ and ⊥ means parallel and perpendicular to the laser beam scanning direction.

## Data Availability

The data are available upon request to the authors.

## Supplementary Materials

The following supporting information can be downloaded at: https://www.mdpi.com/article/10.3390/mi17060653/s1, Figure S1: Variation of IR radiation intervals during the AAT process: (a) Max S235 sample; (b) Scale S235 sample; Figure S2: Variation of IR radiation intervals during the AAT process: (a) Body S235 sample; (b) Body AISI304 sample; Figure S3: Variation of IR radiation intervals during the AAT process: (a) Max AISI 304 sample; (b) Corrosion S235 sample; Figure S4: Variation of IR radiation intervals during the BAT process: (a) Max AISI 304 sample; (b) Corrosion S235 sample; Figure S5: Variation of IR radiation intervals during the BAT process: (a) Max S235 sample; (b) Scale S235 sample; Figure S6: Variation of IR radiation intervals during the BAT process: (a) Body sub. S235; (b) Body sub. AISI 304; Figure S7: Time-domain responses of sample Body S235 BAT, a zoomed-in view of channel C1 response to a single laser pulse; Figure S8: Comparison of time-domain responses of sample Max S235 for AAT and BAT processes in channel C1 for different repetitions; Figure S9: Time-domain responses of sample Max S235 for AAT process. Full temporal response of channel C1 to two laser pulse sequences; Figure S10: Histogram of IR radiation intervals during the AAT process: Max S235 sample, (channel C2); Figure S11: Emissivity measurement for all samples; Figure S12: EDS analysis of the paint before laser irradiation; Figure S13: EDS analysis of the paint after 1x repetition at AAT conditions.
