# Supplementary material for: Fast Thermal Monitoring of Pulsed Laser Cleaning Processes
Source: Micromachines (Basel). 2026 May 25;17(6):653. doi: 10.3390/mi17060653 (PMC13303525; doi:10.3390/mi17060653)
Supplement: Supplementary file 1 [file micromachines-17-00653-s001.zip › micromachines-4259819-supplementary.pdf]

Supplementary Materials

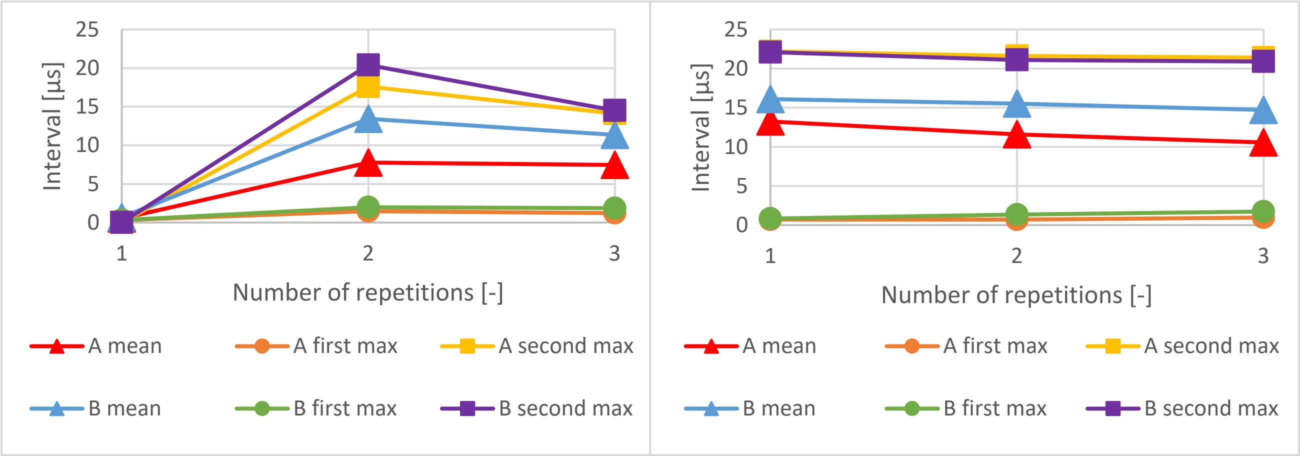

(**a**) (**b**)

Figure S1. Variation of IR radiation intervals during the AAT process: (**a**) Max S235 sample; (**b**) Scale S235 sample.

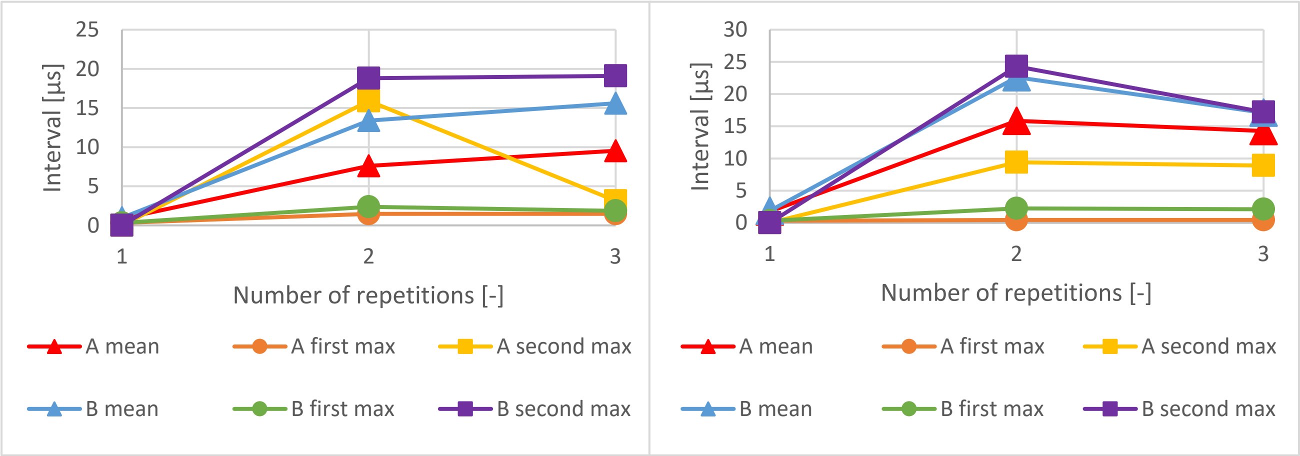

(**a**) (**b**)

Figure S2. Variation of IR radiation intervals during the AAT process: (**a**) Body S235 sample; (**b**) Body AISI304 sample.

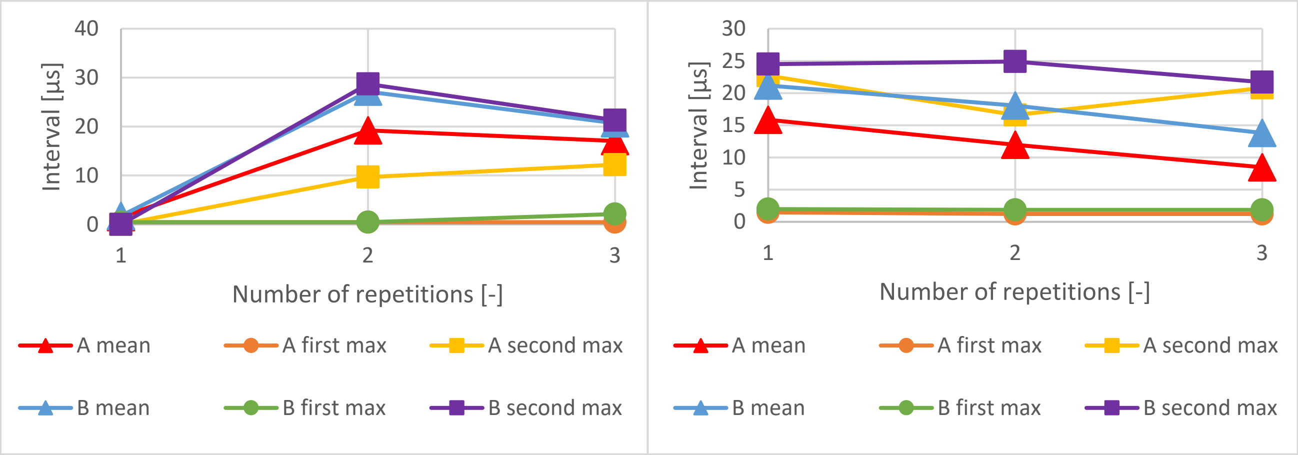

(**a**) (**b**)

Figure S3. Variation of IR radiation intervals during the AAT process: (**a**) Max AISI 304 sample; (**b**) Corrosion S235 sample.

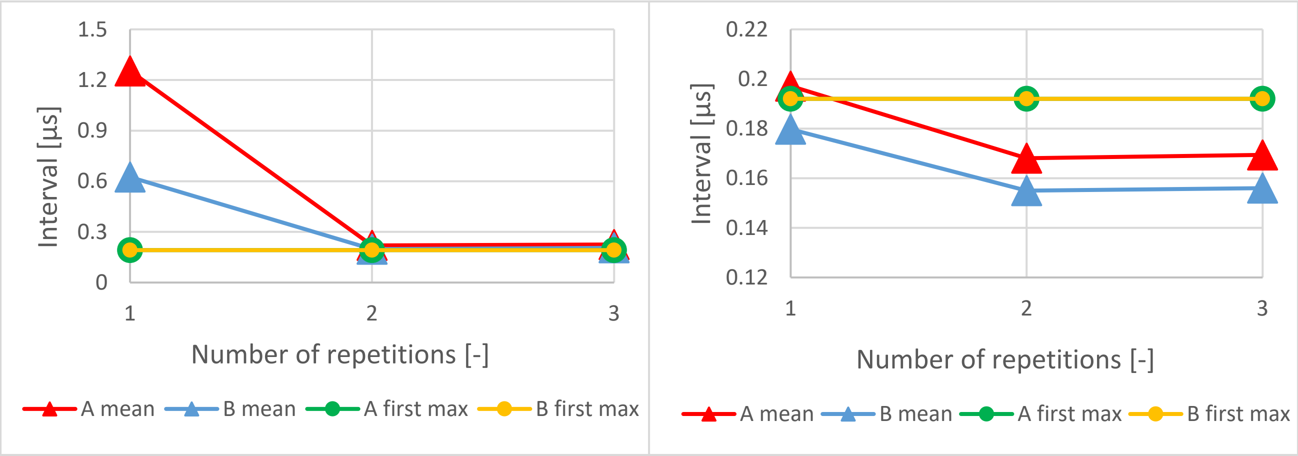

(**a**) (**b**)

Figure S4. Variation of IR radiation intervals during the BAT process: (**a**) Max AISI 304 sample; (**b**) Corrosion S235 sample.

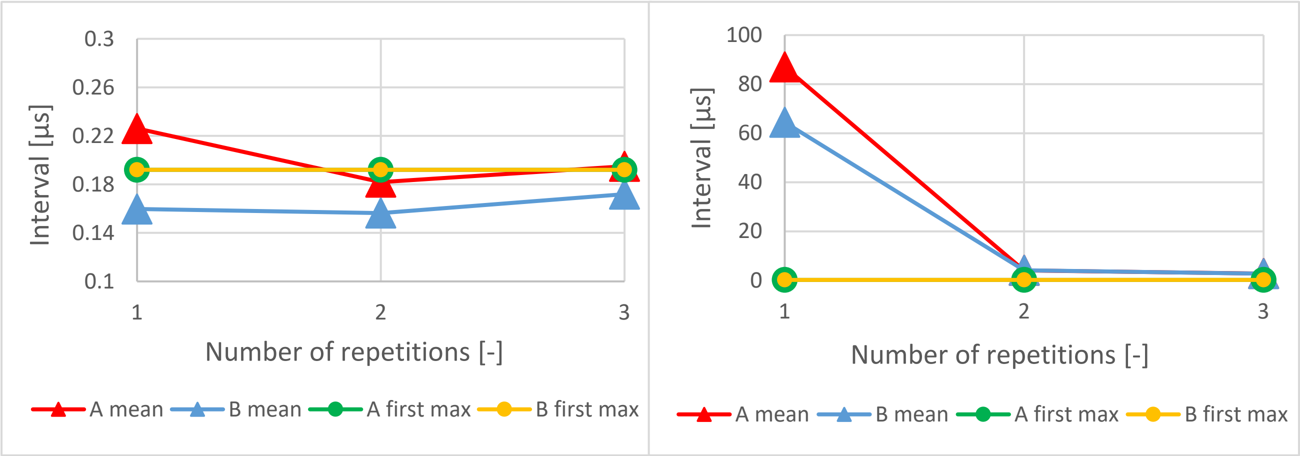

(**a**) (**b**)

Figure S5. Variation of IR radiation intervals during the BAT process: (**a**) Max S235 sample; (**b**) Scale S235 sample.

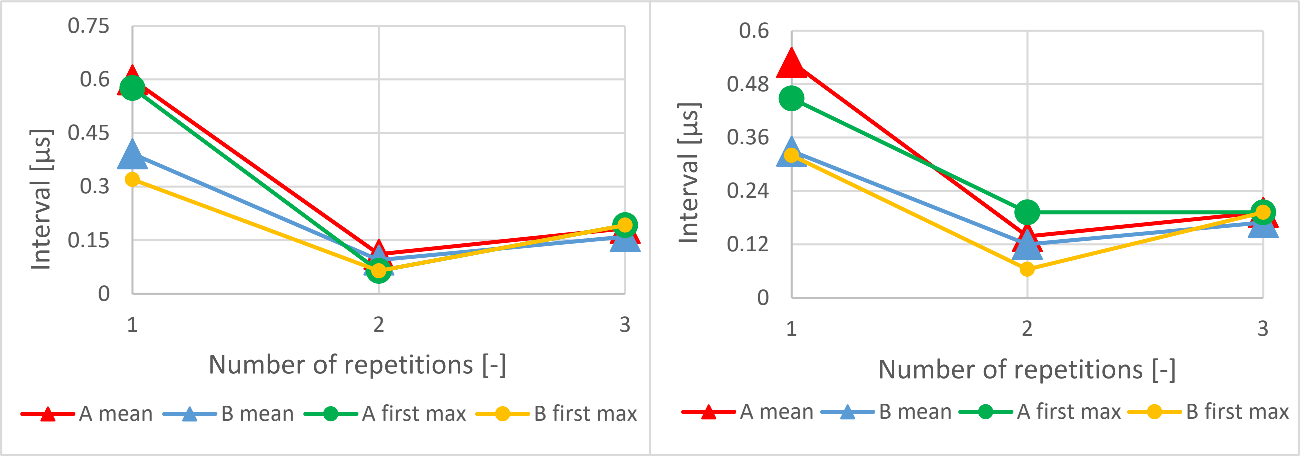

(**a**) (**b**)

Figure S6. Variation of IR radiation intervals during the BAT process: (**a**) Body sub. S235; (**b**) Body sub. AISI 304.

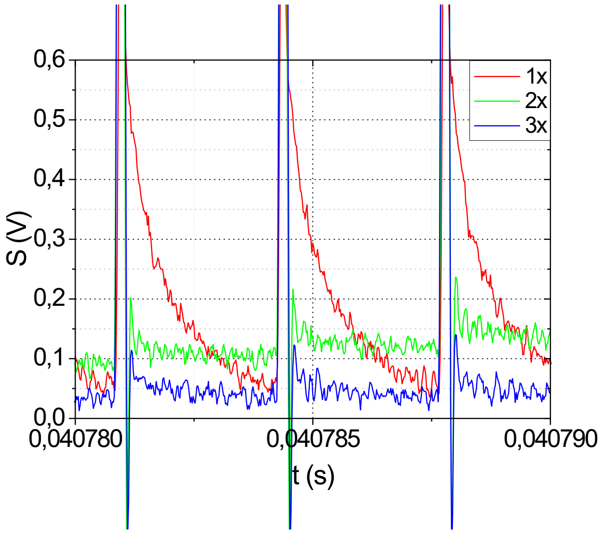

Figure S7. Time-domain responses of sample Body S235 BAT, a zoomed-in view of channel C1 response to a single laser pulse.

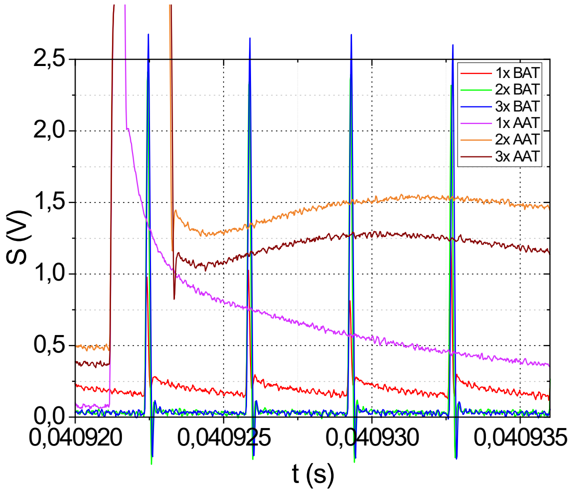

Figure S8. Comparison of time-domain responses of sample Max S235 for AAT and BAT processes in channel C1 for different repetitions.

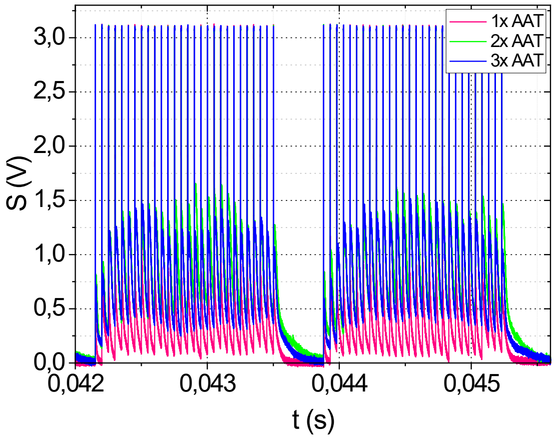

Figure S9. Time-domain responses of sample Max S235 for AAT process. Full temporal response of channel C1 to two laser pulse sequences.

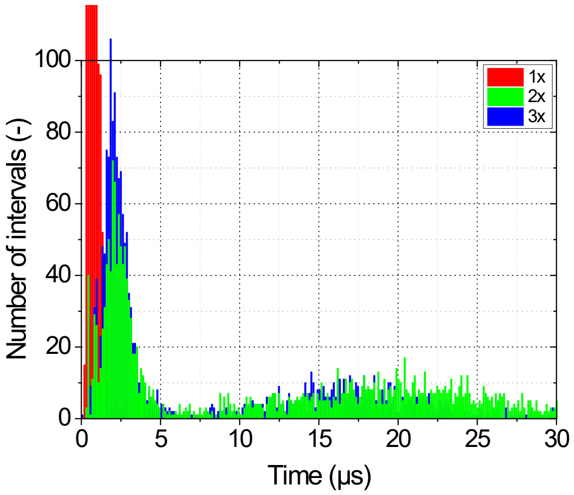

Figure S10. Histogram of IR radiation intervals during the AAT process: Max S235 sample, (channel C2).

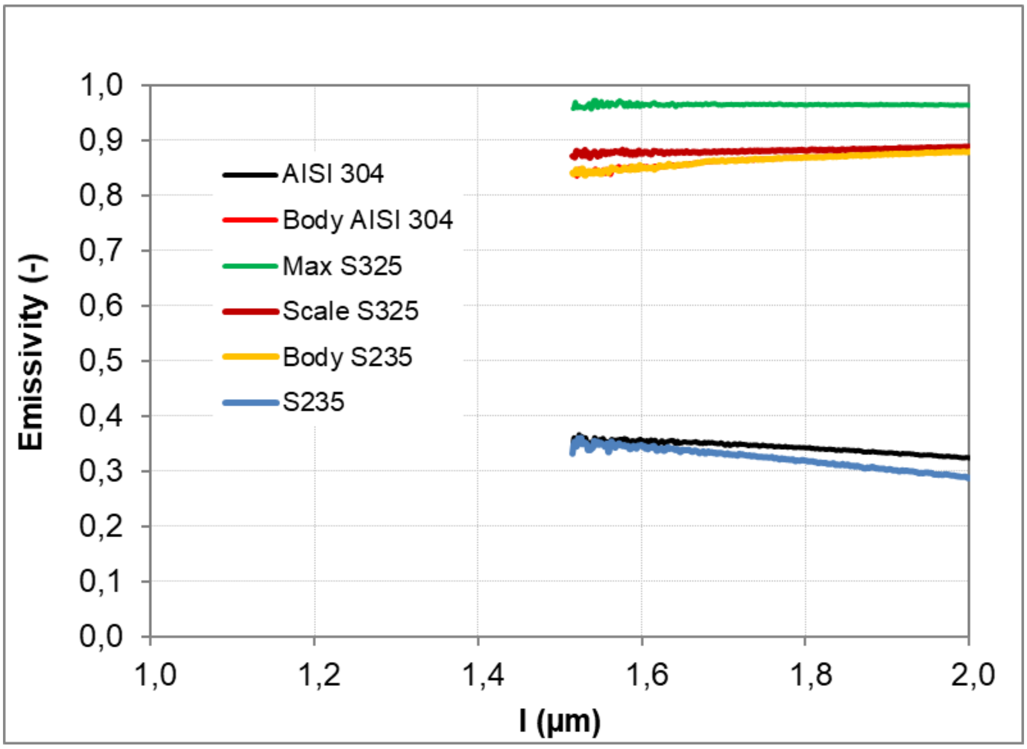

Figure S11. Normal spectral emissivity for different samples determined indirectly from normal hemispherical reflectivity measured at room temperature using an integrating sphere and a calibrated reflection standard.

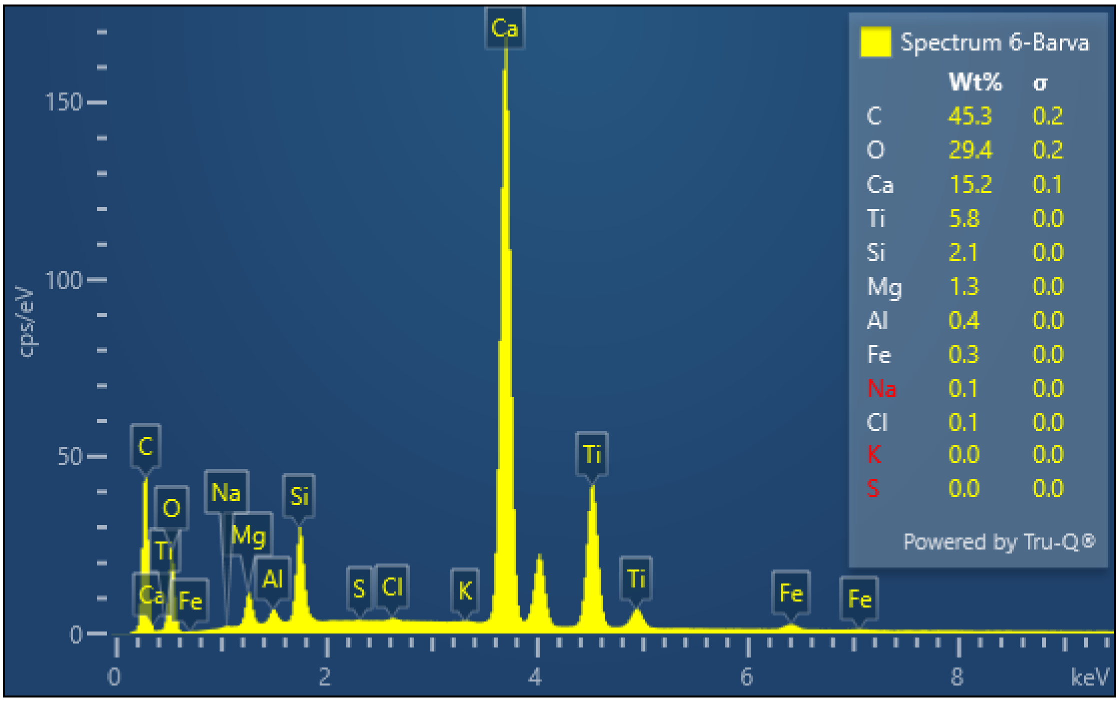

Figure S12. EDS analysis of the paint before laser irradiation.

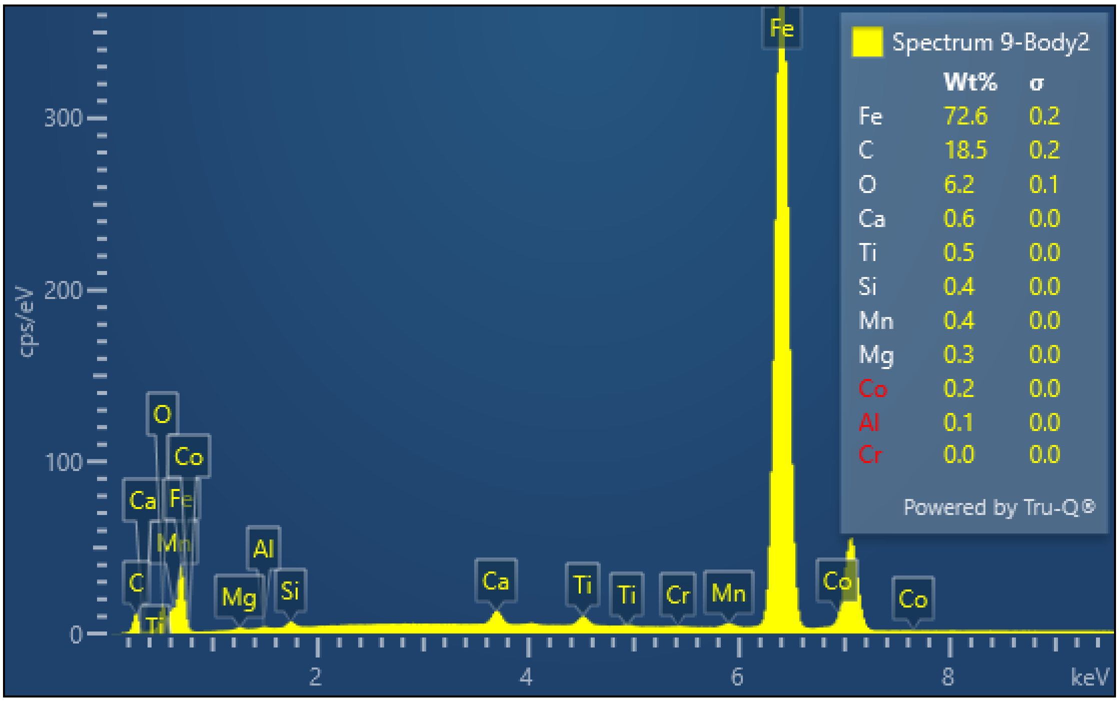

Figure S13. EDS analysis of the paint after 1x repetition at AAT conditions.
